# Tangential Flow Filtration for the Concentration of Oncolytic Measles Virus: The Influence of Filter Properties and the Cell Culture Medium

**DOI:** 10.3390/membranes9120160

**Published:** 2019-11-29

**Authors:** Daniel Loewe, Tanja A. Grein, Hauke Dieken, Tobias Weidner, Denise Salzig, Peter Czermak

**Affiliations:** 1 Institute of Bioprocess Engineering and Pharmaceutical Technology, University of Applied Sciences Mittelhessen, Wiesenstraße 14, 35390 Giessen, Germany; daniel.loewe@lse.thm.de (D.L.); tanjagrein@gmx.de (T.A.G.); hauke.dieken@lse.thm.de (H.D.); t_weidner@mail.de (T.W.); denise.salzig@lse.thm.de (D.S.); 2 Faculty of Biology and Chemistry, University of Giessen, 35390 Giessen, Germany; 3 Fraunhofer Institute for Molecular Biology and Applied Ecology (IME), Project Group Bioresources, Winchesterstr. 3, 35394 Giessen, Germany

**Keywords:** flat-sheet membrane, serum-containing medium, serum-free medium, membrane fouling, oncolytic virus, mathematical modeling, polyether sulfone, cross-linked regenerated cellulose

## Abstract

The therapeutic use of oncolytic measles virus (MV) for cancer treatment requires >10^8^ infectious MV particles per dose in a highly pure form. The concentration/purification of viruses is typically achieved by tangential flow filtration (TFF) but the efficiency of this process for the preparation of MV has not been tested in detail. We therefore investigated the influence of membrane material, feed composition, and pore size or molecular weight cut-off (MWCO) on the recovery of MV by TFF in concentration mode. We achieved the recovery of infectious MV particles using membranes with a MWCO ≤ 300 kDa regardless of the membrane material and whether or not serum was present in the feed. However, serum proteins in the medium affected membrane flux and promoted fouling. The severity of fouling was dependent on the membrane material, with the cellulose-based membrane showing the lowest susceptibility. We found that impurities such as proteins and host cell DNA were best depleted using membranes with a MWCO ≥ 300 kDa. We conclude that TFF in concentration mode is a robust unit operation to concentrate infectious MV particles while depleting impurities such as non-infectious MV particles, proteins, and host cell DNA.

## 1. Introduction

Oncolytic viruses are a promising class of therapeutics for the treatment of late-stage cancer because they specifically infect and lyse cancer cells [1]. Measles virus (MV) is a leading candidate owing to the excellent safety profile of the measles vaccine [2]. However, at least 10^8^ infectious MV particles are needed per dose for cancer therapy [3,4] and this has prompted extensive studies focusing on the optimization of upstream processes [5,6,7,8,9,10]. The deeper understanding of MV production gained from these studies has shown that shear stress and aeration play a key role in process productivity [7]. By optimizing these parameters, MV titers of up to 10^10^ TCID_50_ mL^−1^ have been produced in bioreactors [7]. However, the higher upstream productivity has shifted the focus to downstream processing (DSP), which must concentrate the MV particles and purify them according to regulatory guidelines. This requires the depletion of impurities such as host cell proteins (typical threshold: 100 ng mL^−1^) and host cell DNA (typical threshold applied to vaccines: 10 ng per dose) [11,12,13], as well as the removal of animal components, where a maximum total content of 50 ng per dose bovine serum albumin (BSA) is allowed [14,15].

MV particles are large (~350 nm, range 50–1000 nm [16]) and complex structures, and their inherent instability demands well-chosen DSP strategies [9,17,18]. However, few MV studies have focused on DSP. Early experiments involved MV production in serum-containing medium (SCM), usually with 2–7% (*v*/*v*) fetal bovine serum (FBS), and used 100-kDa tangential flow filtration (TFF) membranes for volume reduction before purification by centrifugation. These studies focused on volume reduction rather than the characteristics of TFF, and provided data for virus recovery (62–100%) and in some cases the reduction of host cell protein levels [19,20]. After decades without further progress, the advent of oncolytic MV therapy has rekindled interest in the DSP steps, including the concentration/purification of MV produced in serum-free medium (SFM) [18,21]. MV purification is not trivial, and significant losses are often reported: up to 42% during microfiltration, 79–84% during centrifugal ultrafiltration, 83% during ion-exchange chromatography, and 40% during hydrophobic interaction chromatography [18,21]. The influence of the cell culture medium on DSP has not been investigated.

TFF may be beneficial for the concentration/purification of oncolytic MV because it is highly scalable (true linear scalability for flat-sheet membranes), much less expensive than chromatography, and has already been used with success for many other viral products [22,23,24,25,26,27,28]. However, membrane-based processes must be investigated carefully to determine the influence of key process parameters such as the membrane material and pore size/molecular weight cut-off (MWCO), both of which affect product purity and recovery [29,30,31,32]. Such investigations can help to ensure the selection of an appropriate type of membrane [23,30,33,34]. Organic membranes for TFF are typically made of cross-linked regenerated cellulose (xRC), polysulfone, or polyether sulfone (PES), although other materials such as polyvinylidene fluoride may also be suitable.

Membrane filtration is a pressure-driven process that generally follows Darcy’s law, where the flux through a membrane is dependent on the transmembrane pressure (TMP), the fluid viscosity, and the total membrane resistance (Equation (1)):

(1) $$J=\frac{1}{A}\cdot \frac{\mathrm{dV}}{\mathrm{dt}}=\frac{\mathrm{TMP}}{µ\cdot R_{\mathrm{total}}}$$

Accordingly, the TMP is the main driving force generating the pressure difference between the retentate (inflow) and permeate (outflow) sides. Due to the dynamic flow along the membrane on the retentate side, the retentate pressure is the mean pressure of the inlet and outlet flow of the membrane. Mathematically this is expressed as shown in Equation (2):

(2) $$\mathrm{TMP}=\frac{p_{f}+p_{r}}{2}-p_{p}$$

The overall permeate flux is also influenced by the total filtration resistance ($R_{\mathrm{total}}$), which can be calculated as a series of separate resistances using the resistance-in-series model (Equation (3)). At least two types of resistance are considered: the resistance of the pure membrane and the resistance caused by membrane fouling (e.g., due to protein deposition). The fouling resistance can in turn be separated in various categories, including reversible and irreversible fouling.

(3) $$R_{\mathrm{total}}=R_{m}+R_{f}$$

The resistance-in-series model is useful to distinguish between components that influence membrane fouling and/or cleaning, but it does not allow direct conclusions to be drawn about the fouling mechanism. The predominant fouling mechanism in a filtration experiment can be described in terms of the permeate volume (V) and the filtration time under constant pressure conditions [35] as shown in Equation (4):

(4) $$\left(\frac{d^{2}t}{{\mathrm{dV}}^{2}}\right)=k\cdot {\left(\frac{\mathrm{dt}}{\mathrm{dV}}\right)}^{m}$$

Fouling may involve any of four dominant mechanisms [35]: complete blocking, pore blocking, intermediate blocking, and cake filtration (Table 1). These mechanisms are represented by different values of m in Equation (4). By fitting experimental data to these models, the predominant fouling mechanism can be determined.

To investigate the TFF concentration process for MV in more detail, we looked at the relationship between the concentration of infectious MV and the use of two different membrane materials with a range of pore sizes/MWCOs. Accordingly we tested four membranes: three made from PES (100-nm pore size, 300-kDa and 100-kDa MWCO) and one from xRC (Hydrosart, 100 kDa MWCO). We also determined the impact of switching from SCM to SFM. We have previously reported that MV upstream titers are not affected by changing the medium [8] but the effect of medium composition on DSP has not been reported. We analyzed the flux behavior during TFF in concentration mode, the recovery of infectious MV, and determined whether major impurities (non-infectious MV, protein, and host cell DNA) were depleted (although this was not the primary aim of our TFF approach). Finally, to achieve a deeper understanding of the fouling mechanisms that affect MV concentration, we fitted our data to the corresponding mathematical models [35]. A well-chosen membrane leads to the excellent recovery of infectious MV while also clearing impurities such as non-infectious MV, protein, and host cell DNA. Furthermore, a high flux is recommended during TFF to achieve an economic unit operation as part of the complete DSP train. 

## 2. Materials and Methods

### 2.1. Membranes

Four different organic membranes were tested. All of them were flat-sheet membranes (Slice200) with a filtration area of 0.02 m², and were manufactured by Sartorius Stedim Biotech (Göttingen, Germany). Three of the tested membranes (pore size = 100 nm, MWCO = 300 kDa and 100 kDa) were manufactured from PES and the other (MWCO = 100 kDa) from xRC (Hydrosart, Sartorius Stedim Biotech, Göttingen, Germany).

### 2.2. Cell Culture and MV Propagation

The MV strain MVvac2 GFP (P) was kindly provided by Dr. Michael Muehlebach (Paul-Ehrlich-Institute, Langen, Germany). The dynamic production of MV in suspension was carried out in a stirred-tank reactor [6,8]. Vero cells (CCL-81, ATCC) were grown on 3 g L^−1^ Cytodex 1 microcarriers (GE Healthcare, Chicago, Illinois, USA) in SCM or SFM. They were allowed to attach to the microcarriers for ~4 h before the addition of MV at a multiplicity of infection of 30 [8]. The SCM was DMEM-HG (Bichrom, Berlin, Germany) supplemented with 10% (v/v) FBS (Biochrom) and 10 mM HEPES (pH 7.4). The SFM was VP-SFM (Thermo Fisher Scientific, Waltham, MA, USA).

### 2.3. Pretreatment of MV Suspensions Prior to TFF

**Figure 1:**
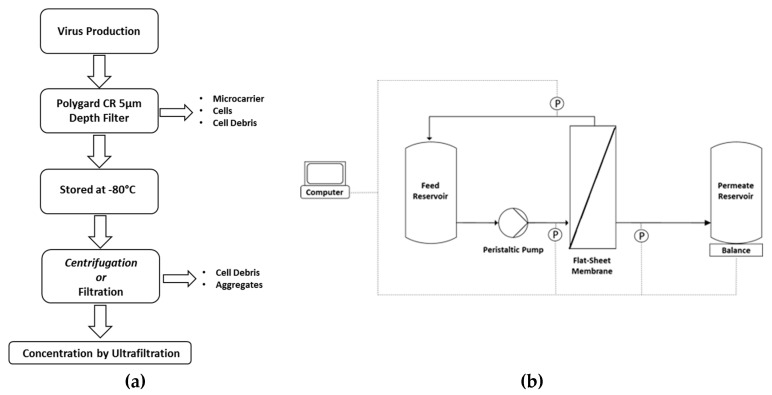
(**a**) Workflow for the processing of measles virus (MV) suspensions before tangential flow filtration experiments. (**b**) Experimental setup. The measles virus suspension was introduced using a Tandem 1082 peristaltic pump at a flow rate of 150 mL min^−1^ with a transmembrane pressure of 0.2 bar. The permeate weight was measured using a digital balance. Pressure was recorded using Sci-Log pressure sensors. Data were analyzed using WinWedge software.

The MV-containing supernatant was harvested from the reactor by passing the broth through a 5-µm Polygard CR Opticap XL-Capsule (Merck Millipore, Burlington, Massachusetts, USA) to remove microcarriers, cells, and cell debris, and was stored at −80 °C. Before TFF experiments, MV suspensions were centrifuged for 10 min at 300× *g* using a Heraeus Multifuge X1R (Thermo Fisher Scientific) or clarified by depth filtration/microfiltration (Figure 1a). The clarified suspensions were pooled before transfer to the TFF system (Figure 1b).

### 2.4. Ultrafiltration in TFF Mode (UF-TFF)

#### 2.4.1. Pure Water Flux Determination

The pure water flux was measured using MilliQ water (Merck, Darmstadt, Germany) at ~20 °C. New membranes were installed in the flat-sheet membrane holder (Sartorius Stedim) and the fluxes were determined at different TMPs (0.2, 0.4, and 0.6 bar) using the constant pressure mode of the Sartorius Sartflow system. Independent experiments were carried out in triplicate. If necessary the tube on the retentate side was clamped to achieve the desired TMP. Permeate flux was measured on a Sartorius digital balance after showing no variation >1 mL for several minutes.

#### 2.4.2. TFF in Concentration Mode

For the TFF experiments, the membranes were flushed by circulation (closed permeate valve) with 400 mL of 1 M NaOH (40 °C, 1 h). After measuring the clean water flux with MilliQ water, the membranes were equilibrated with 100 mL of 20 mM Tris-HCl (pH 7.4). The feed reservoir was then filled with 450 mL of MV suspension in SCM or SFM. 

The feed was circulated for the first 5 min with a closed permeate outlet. The permeate outlet was then opened and we recorded the retentate, permeate and feed pressures using SciLog pressure sensors (Parker Hannifin, Cleveland, OH, USA), and the permeate weights using a Sartorius digital balance. The data were analyzed using WinWedge software. Samples of ~0.8 mL were taken from the feed and permeate vessels for each 50 g of permeate that passed the membrane (Figure 1). The filtration run was stopped when the permeate weight reached 420 g (concentration factor = 15). After each filtration run, the feed vessel was emptied and the membrane was flushed with pure water until a constant permeate flux was achieved. Before the next filtration run, the membrane was incubated for 40–48 h in 1 M NaOH (40 °C).

### 2.5. Determination of Fluid Viscosity

The viscosity of the permeate fractions (2 mL) was measured at 20 °C using a Haake viscosimeter (Thermo Fisher Scientific) with cone-plate geometry.

### 2.6. Assays

#### 2.6.1. MV Infectivity Assay

MV infectivity was determined using the TCID_50_ method [8]. Vero cells were seeded into clear 96-well plates (Sarstedt, Germay) at a concentration of 50,000 cells/mL in DMEM supplemented with 2 mM L-glutamine and 10% (*v/v*) FBS. We transferred 200 µL to each well and incubated the plates at 37 °C in an 8% CO_2_ atmosphere for at least 4 h. MV fractions for analysis were serially diluted 1:10 in a separate 96-well plate (10^−1^ to 10^−12^, total volume 300 µL) in the same medium. The MV dilution series was then transferred in 30-µL aliquots to the 96-well plate containing Vero cells and incubated for 7 days at 32 °C in an 8% CO_2_ atmosphere. MV infection was then recorded by fluorescence microscopy (Hund, Germany) and the titer was determined as previously described [36,37].

#### 2.6.2. Determination of Total Virus RNA Levels

Total virus RNA was extracted using the Quick-RNA viral kit (Zymo Research, Irvine, California, USA) and the amount was determined by real-time quantitative polymerase chain reaction (RT-qPCR) with SYBR staining according to the manufacturer’s recommendations (BioLine, Luckenwalde, Germany). The target MV sequence was amplified using forward primer 5′-TGG CAT CTG AAC TCG GTA TCA C-3′ and reverse primer 5′-TGT CCT CAG TAG TAT GCA TTG CAA-3′ (Appendix A). The target MV sequence was reverse transcribed for 10 min at 45 °C and the reaction was heated to 95 °C for 2 min to activate the enzyme before 40 cycles of 95 °C for 15 s, 55 °C for 15 s, and 68 °C for 10 s. The specificity of the product was confirmed by melt curve analysis. A synthetic *N* gene sequence was used to construct a standard curve to determine the total amount of viral RNA. The detection limit was 10^3^ copies per µL. We assumed that one copy represents one virus particle, but free viral RNA is also measured with this assay.

#### 2.6.3. Determination of the Total Protein Content 

Total protein levels were determined in clear flat-bottom 96-well plates (Greiner Bio-One, Kremsmünster, Austria) using the Pierce BCA assay kit (Thermo Fisher Scientific) and BSA for calibration. The absorbance was measured at 562 nm in a Cytation 3 microplate reader (Biotek, Winooski, Vermont, USA). The detection limit was 25 µg mL^−1^.

#### 2.6.4. Determination of the Host Cell DNA Content

The content of dsDNA was determined using the Quant-iT Picogreen reagent (Thermo Fisher Scientific) with bacteriophage λ DNA as a standard. The assay was carried out in black flat-bottom 96-well microplates (Nunc, Roskilde, Denmark) and the fluorescence (λ_excitation_ = 485 nm, λ_emission_ = 535 nm) was measured in the Cytation 3 microplate reader. The detection limit was 1 ng mL^−1^.

### 2.7. Calculations

#### 2.7.1. Hydraulic Permeability and Permeability of the Suspension Components

The hydraulic permeability (L_P_) of each membrane was calculated based on the permeate flux of water (20 °C) at different TMPs (Equation (5)):

(5) $$L_{P}=\frac{J_{w}}{\mathrm{TMP}}$$

where $J_{w}$ is the clean water flux.

The permeability (α) of a molecular species was defined as the ratio of the concentration in the initial feed ($c_{\mathrm{feed}}$) to the concentration in the permeate fraction ($c_{\mathrm{permeate}}$) as shown in Equation (6):

(6) $$\propto =\frac{c_{\mathrm{permeate}}}{c_{\mathrm{feed}}}$$

The closer the number is to 1, the more suitable the membrane is for purification by diafiltration or infinite concentration.

#### 2.7.2. Productivity of a Filtration Run

The feed processed per unit membrane and time is defined as productivity (Equation (7)):

(7) $$\mathrm{Productivity}=\frac{V_{\mathrm{permeate}}}{A_{\mathrm{membrane}}\cdot t_{\mathrm{filtration}}}$$

#### 2.7.3. Flux Recovery

The flux recovery is defined as the pure water flux after MV filtration relative to the pure water flux of the clean membrane (Equation (8)). The higher this value, the lower is the prevalence of irreversible fouling on the membrane surface.

(8) $$J_{\mathrm{recovery}}=\frac{J_{\mathrm{rf}}}{J_{0}}$$

where $J_{\mathrm{rf}}$ is the pure water flux after the filtration experiment and $J_{0}$ is the pure water flux of the clean membrane.

#### 2.7.4. Product Recovery and Impurity Clearance

The error of the infectious virus titer is high (~0.5 log_10_) so the change in virus titer was calculated and expressed as the logarithmic fold change (Equation (9)):

(9) $${\mathrm{Log}}_{10}\;\mathrm{fold}\;\mathrm{change}=\log_{10}\left(\frac{c_{\mathrm{retentate}}}{c_{\mathrm{feed}}}\right)$$

where $c_{\mathrm{retentate}}$ is the infetious virus titer in the retentate and $c_{\mathrm{feed}}$ fraction.

The relative recovery of total viral RNA was calculated as shown in Equation (10):

(10) $$\mathrm{Relative}\;r\mathrm{ecovery}=\;\frac{c_{i}}{c_{0}\cdot \mathrm{cf}}$$

where c_0_ is the initial concentration and c_i_ is the concentration corresponding to the concentration factor (cf).

The relative clearance of impurities such as total proteins and host cell DNA was calculated as shown in Equation (11):

(11) $$\mathrm{Relative}\;\mathrm{clearance}\;=1-\;\frac{c_{i}}{c_{0}\cdot \mathrm{cf}}$$

#### 2.7.5. Rating of Experimental Data Fitting to Fouling Models

The experimental data were fitted to the fouling models [34] in Matlab, and the best fit between the experimental data and the modeled fouling function was estimated as the minimal sum square error (SSE) using the function “fminsearch” according to Equation (12).

(12) $$\mathrm{SSE}=\;\sum {\left(y_{i}-\hat{y_{i}}\right)}^{2}$$

We evaluated the fitting and thus found the predominant fouling mechanism by calculating the R^2^ as shown in Equation (13):

(13) $$R_{\mathrm{fit}}{}^{2}=1-\frac{\sum {\left(y_{i}-\hat{y_{i}}\right)}^{2}}{\sum {\left(y_{i}-\bar{y_{i}}\right)}^{2}}$$

### 2.8. Statistical Analysis

Statistically significant differences were identified using Student’s *t*-test. 

## 3. Results

To develop a TFF-based process for the concentration of MV, we tested four membranes manufactured from two different materials (PES and xRC) with MWCOs of ~1000 kDa (pore size 100 nm), 300 kDa, and 100 kDa. These MWCOs were selected according to the typical size of MV particles (~350 nm [17], range 50–900 nm [16]). A MWCO of ~1000 kDa should therefore retain most MV particles, but smaller MWCOs may be needed to account for the heterogeneous size distribution. We also investigated the influence of the cell culture medium on the process performance.

### 3.1. Characterization of the Membrane and the Feed Solution

#### 3.1.1. Characterization of the Membrane Flux

**Figure 2:**
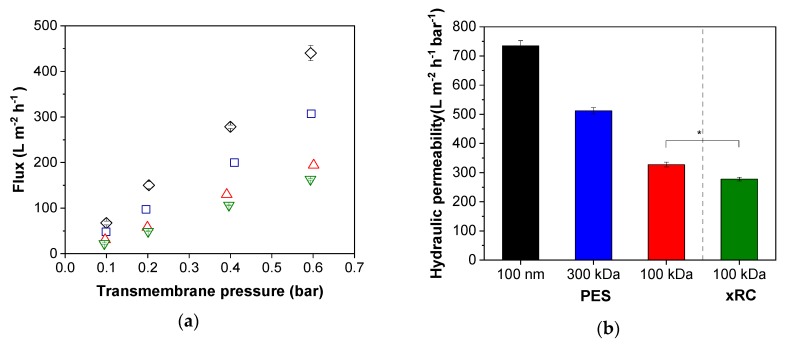
Comparison of membrane performance. (**a**) Changes in flux according to the transmembrane pressure (black = 100 nm, blue = 300 kDa, red = 100 kDa polyether sulfone (PES), green = 100 kDa cross-linked regenerated cellulose (xRC)). (**b**) Hydraulic permeability of the membranes. In each panel, the values are means ± standard deviations (*n* = 3). Significant differences were observed between the 100-kDa xRC membrane and all other membranes, but for clarity we only represent the difference between the two 100-kDa membranes (* *p* < 0.05).

For initial characterization, we determined the TMP-dependent pure water flux of each membrane. The water flux and thus the hydraulic permeability decreased for PES membranes with lower MWCO values (Figure 2). The comparison of the different membrane materials revealed a slightly lower TMP-dependent increase in the pure water flux and therefore a higher membrane resistance (Equation (1)) for the xRC membrane. If we consider the pure water flux experiments alone, we would choose the PES membrane with a pore size of 100 nm because this achieved the highest hydraulic permeability. However, the pure water flux does not necessarily predict a membrane’s performance when filtering the cell culture supernatant to recover infectious MV particles.

#### 3.1.2. Characterization of the Feed

The system was fed with MV-containing supernatants based on a medium containing FBS (SCM) or a commercial SFM. We determined the quantity of infectious MV particles and total viral RNA as well as the abundance of key impurities (protein and host cell DNA) as shown in Table 2. We also calculated the ratio of total to infectious MV particles (R_T/I_) which is an important parameter in MV downstream processes. The total virus RNA and the R_T/I_ were similar for MV produced in SCM and SFM. The DNA content was also similar, but the total protein content was 87.2% lower when MV was produced in SFM, reflecting the absence of serum proteins.

### 3.2. Concentration of MV

Having characterized the membranes and the feed, we tested all eight membrane/feed combinations and concentrated MV particles by TFF at a constant TMP of 0.2 bar. We selected the lowest TMP that could be applied to each membrane, given the shear-sensitive nature of MV [17]. For effective concentration, the membrane must retain the infectious virus, ideally allowing the impurities through to the permeate side. Initially, we investigated the recovery of infectious MV particles and total viral RNA for all four membranes. It is important to recover infectious particles because only these are suitable for oncolytic therapy. Therefore, the filtration process should not inactivate the virus.

#### 3.2.1. Virus Recovery during TFF

**Figure 3:**
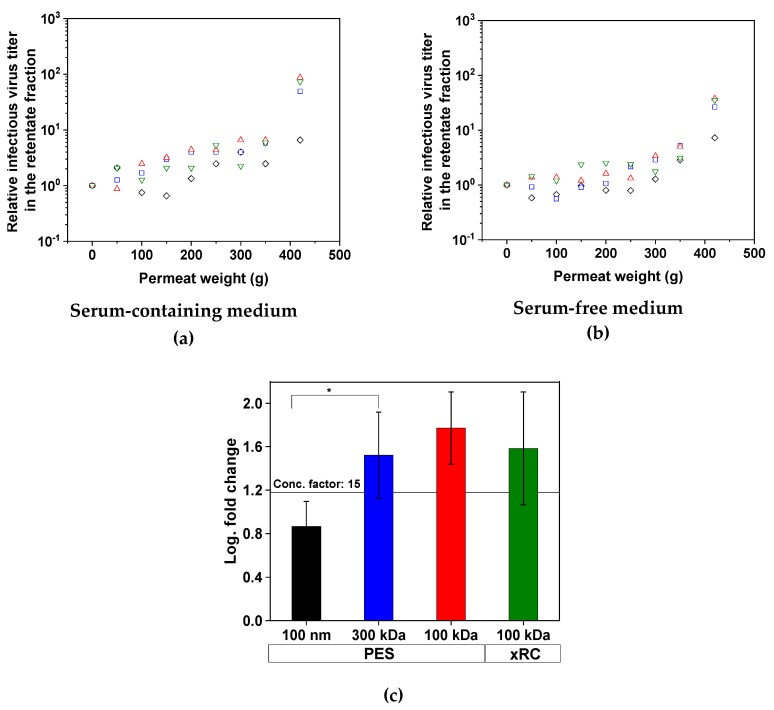
Concentration of infectious MV particles in the retentate fraction during tangential flow filtration. The data points show the relative infectious MV titer in the retentate fraction correlating with the permeate weight. Therefore, with increasing permeate weight, the retentate volume decreases and the MV titer increases. For more clarity, the mean of the two separate concentration experiments for each membrane is plotted for the process using (**a**) SCM and (**b**) SFM. (**c**) Summary of the four filtration runs, indicating the full recovery of MV for the 300-kDa (blue) and 100-kDa membranes (PES = red, xRC = green). The 100-nm membrane (black) achieved a lower recovery. A concentration factor of 15 is equivalent to a log fold-change of 1.18). Values are means ± standard deviations (*n* = 4). Significant differences were observed between the 100-nm PES membrane and all other membranes, but for clarity we only represent the difference between the 100-nm and 300-kDa membranes (* *p* < 0.05).

The concentration of infectious MV particles in the retentate increased during filtration for all four membranes, meaning that an effective concentration process was achieved in all cases (Figure 3). The concentration factor was at least 15 for three of the membranes, corresponding to a log fold-change of 1.18, but the 100-nm PES membrane only achieved a concentration factor of 7.3 (*p < 0.05). The concentration of MV was not affected by the presence or absence of serum in the medium. All membranes with a MWCO ≤ 300 kDa were able to retain infectious MV particles and the membrane material had no influence on this outcome. In all experimental runs, no infectious MV was found in the permeate.

**Figure 4:**
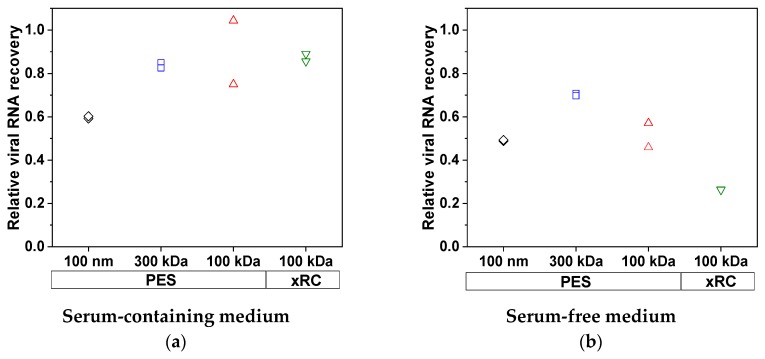
Recovery of total viral RNA from (**a**) SCM and (**b**) SFM. The greatest loss of total virus RNA in SCM was ~40%, as observed for the 100-nm membrane (black), whereas the other membranes showed losses of ~20%. When the MV was produced in SFM, the minimum loss was ~30%, as observed for the 300-kDa membrane (blue). The 100-nm and 100-kDa PES membranes (black and red, respectively) showed similar losses of ~50%. The xRC membrane (green) showed much higher losses than the PES membranes (~75%). The experiments were conducted in duplicate (*n* = 2), and each result is presented as a single data point.

We also measured the total viral RNA content, which represents the sum of infectious and non-infectious viruses as well as free RNA released from disrupted virus particles. Ideally, the RNA content must be reduced to the level that is expected for the infectious particles. More total viral RNA was lost when MV was produced in SFM (Figure 4) but the lost RNA was not found in the permeate (<0.01% of total RNA was found in the permeate), indicating that it must bind to the membrane. The 100-nm membrane achieved only 59.2% and 49.0% recovery of total RNA from SCM and SFM, respectively. At lower MWCOs, the recovery of MV from SCM increased to a constant high value of 83.7–89.7%, which was not improved by decreasing the MWCO or changing membrane material. With the 300-kDa membrane, we achieved a recovery of 70.2% from the SFM, but this declined to 51.5% (PES) and 26.4% (xRC) with the 100-kDa membranes, indicating that more RNA/total virus adsorbs to the membrane surface. The xRC membrane therefore clearly adsorbed more total RNA than the PES membrane when the MV was prepared in SFM. Interestingly, the loss of total virus RNA and particles by adsorption to the 100-nm membrane definitely included infectious viruses (given the loss of infectivity discussed above, Figure 3c), whereas the other membranes selectively absorbed non-infectious particles, preserving the high infectivity of the concentrated fraction. 

#### 3.2.2. Removal of Impurities by TFF

Oncolytic MV must be prepared in a highly purified form for therapeutic applications to prevent toxicity and immunogenicity triggered by host cell or culture medium components [38,39,40]. The main impurities are host cell proteins and DNA, as well as proteins from serum (if used). Although purification is not the primary aim of the TFF-based concentration step, it is advantageous if these impurities are cleared.

**Figure 5:**
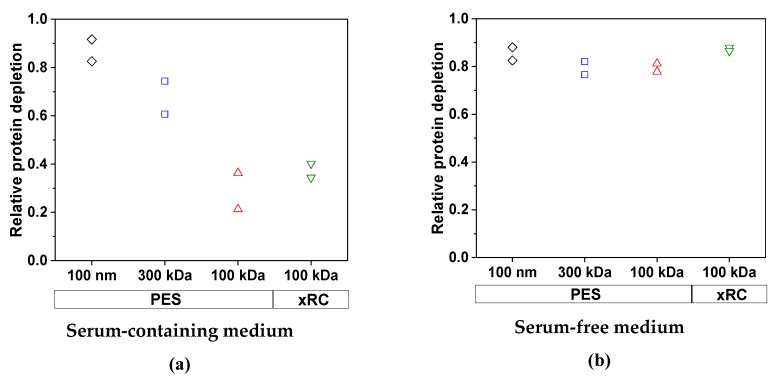
The relative clearance of total proteins by each membrane in (**a**) SCM and (**b**) SFM for an MV concentration factor of 15. The experiments were conducted in duplicate, and each experiment is presented as an independent data point (*n* = 2).

We first investigated the ability of the membranes to reduce the protein content of the feed. As anticipated, the cell culture medium had a significant impact on the removal of total protein (Figure 5). For the MV produced in SFM, the efficiency of protein clearance was similar for all four membranes: 85.3% (100 nm PES), 79.3% (300 kDa PES), 79.5% (100 kDa PES), and 87.2% (100 kDa xRC). We did not observe any effect attributable to the membrane material or pore size/MWCO. In contrast, for the MV produced in SCM, the efficiency of protein clearance was lower and strongly dependent on the pore size/MWCO. Only the 100-nm PES membrane achieved efficient protein depletion (87.1%), whereas the other membranes concentrated the protein in the retentate. The 300-kDa membrane cleared ~67.5% of the total protein, whereas the 100-kDa membranes cleared 28.8% (PES) and 37.3% (xRC). The concentration of the impurities (proteins in this case) therefore increased strongly along with that of the MV particles, due to the lower permeability of the 100-kDa membranes for proteins (Table 3). The membrane material had a clear effect, with the xRC membrane proving to be 54.3% more permeable to proteins than the PES membrane with the same MWCO.

**Figure 6:**
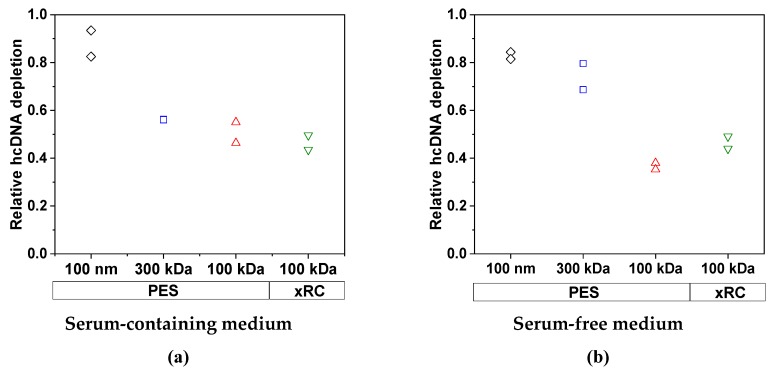
The clearance of host cell DNA by each membrane in (**a**) SCM and (**b**) SFM for an MV concentration factor of 15. The experiments were conducted in duplicate (*n* = 2), and each experiment is presented as an independent data point.

We also measured the clearance of host cell DNA. This was mainly dependent on the pore size/MWCO, whereas the membrane material and the medium (SCM or SFM) played only a minor role. The removal of host cell DNA became less efficient with lower MWCOs as more DNA molecules became trapped by the membrane (Figure 6). The clearance of DNA by the 300-kDa membrane was inhibited in the presence of high concentrations of protein, presumably because the proteins form a gel layer that acts as an additional barrier.

The permeability of the 100-nm membrane for host cell DNA was similar in SCM and SFM, whereas the permeability of the other membranes was slightly lower in SCM compared to SFM, as shown Table 4.

#### 3.2.3. Characterization of the Concentrated MV Fractions

Finally, and with respect to the previous sections, the solute concentrations in the concentrated fractions of each membrane and cell culture medium combination are shown in Table 5.

### 3.3. Analysis of Membrane Fouling

We measured the flux over the permeate weight during the concentration of MV, and used these data to investigate the fouling behavior of the membranes. We used the resistance-in-series model and corresponding equations [35] to identify the dominant fouling mechanism.

#### 3.3.1. Flux during MV Purification

**Figure 7:**
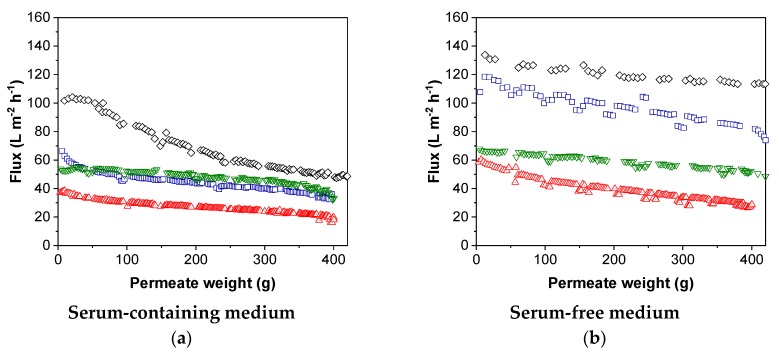
Flux over filtration permeate weight for measles virus (MV) in (**a**) serum-containing (SCM) and (**b**) serum-free medium (SFM). The mean fluxes over permeate weight are presented for the PES membranes with a molecular weight cut-off (MWCO) of ~1000 kDa (100 nm) (black), 300 kDa (blue), and 100 kDa (red), and the xRC membrane with an MWCO of 100 kDa (green).

We concentrated MV by TFF with a constant TMP (0.2 bar) and measured the flux for all four membranes with MV-containing feeds based on SCM or SFM (Figure 7).

We observed a decrease in flux, reflecting the occurrence of fouling which caused the membrane resistance to increase with filtration time. The decrease in flux was dependent on both the pore size/MWCO and the type of medium. The flux in SCM was lower than that in SFM, indicating fouling related to the higher total protein content of SCM. As observed in the pure water experiments, the 100-nm membrane achieved the highest permeate flux in both SCM and SFM. As the MWCO of the PES membrane decreased, so did the flux, regardless of the type of medium. The xRC membrane achieved a higher flux than the PES membrane with the same MWCO.

The flux generally declined with filtration time. In SCM, the flux of the PES membranes initially declined rapidly and then reached a pseudo-steady state. The steepness of the decline in flux was indirectly proportional to the pore size/MWCO. Interestingly, the xRC membrane showed no decline in flux in either medium. For the PES membranes, the decline in flux was steeper in SCM than SFM, but the flux declined throughout the filtration run and did not reach a pseudo-steady state.

The fouling behavior was reflected in the productivity of each membrane (Table 6). The productivity was highest for the 100-nm PES membrane regardless of the medium. As described above for the flux, the productivity declined with decreasing MWCO for the PES membrane regardless of the medium. However, the productivity was up to 2.2-fold higher (300 kDa) in SFM compared SCM. The xRC membrane achieved similar productivities in both media, and outperformed the PES membrane with the same MWCO. In SCM, the xRC membrane even achieved higher productivity than the 300-kDa PES membrane. The 100-kDa xRC membrane therefore appears beneficial for the overall process, because it is less susceptible to fouling and the total protein content has a less severe effect on filtration efficiency.

#### 3.3.2. Determination of Fouling Resistance Using the Resistance-in-Series Model

To calculate the fouling resistance accurately, we determined the viscosities of the permeate fractions, which were higher in SCM than SFM (Table 7). Furthermore, the permeate fractions became less viscous with decreasing MWCO, confirming our finding that more protein remained in the retentate and the membrane became less permeable to proteins.

**Figure 8:**
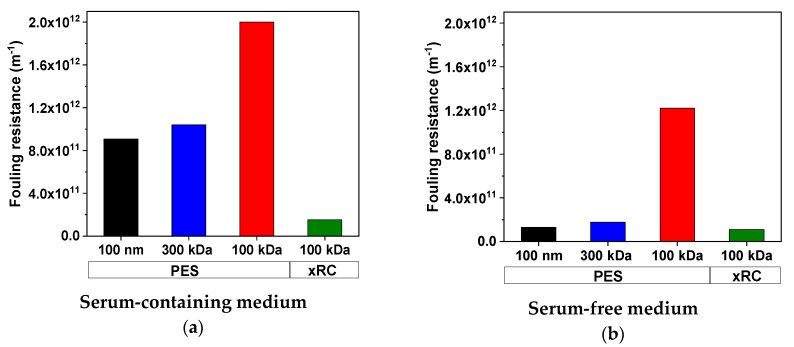
Comparison of fouling resistances for measles virus (MV) in serum-containing medium (**a**) and serum-free medium (**b**). Values are means of two runs (*n* = 2).

Given these viscosities and the empirical permeate fluxes (assuming in all cases a density of ~1 g mL^−1^), we calculated the apparent membrane and fouling resistances using Equations (1) and (3). Due to the inverse relationship between flux and total membrane resistance, lower flux causes higher fouling resistance. The fouling resistances for the 100-nm and 300-kDa PES membranes were similar. Both membranes accumulated a significant fouling layer in SCM (100 nm = 9.07 × 10^11^ m^−1^, 300 kDa = 10.41 × 10^11^ m^−1^) but a weaker layer in SFM (100 nm = 1.31 × 10^11^ m^−1^, 300 kDa = 1.77 × 10^11^ m^−1^). The decrease in fouling resistance was collinear with the decrease in protein content: the protein content of the SFM was 87.2% lower than that of the SCM, and the fouling resistance accordingly decreased by 85.6% (100 nm) and 83.0% (300 kDa) in SFM. The 100-kDa PES membrane showed the highest fouling resistance regardless of the medium (SCM = 20.01 × 10^11^ m^−1^, SFM = 12.22 × 10^11^ m^−1^). In contrast to the PES membranes, the 100-kDa xRC membrane showed a remarkably low fouling resistance (Figure 8).

We found that the fouling was irreversible: flushing of the membrane with water, which should remove reversible fouling, did not increase of permeate flux (data not shown). The relative water flux, measured with pure water after each filtration for all eight membrane/feed combinations (Table 8) therefore confirmed the similar fouling behavior of the 300-kDa and 100-nm membranes, contrasting with the low susceptibility of the xRC membrane. The irreversible fouling layer could only be removed by cleaning. We developed a suitable cleaning strategy (40–48 h in 1 M NaOH at 40 °C) and found that cleaning after filtration could restore the pure water flux of a cleaned membrane to a value similar to that observed before the filtration run (data not shown).

#### 3.3.3. Classification of Fouling Mechanisms

When we encountered fouling resistance, we used the models described above to determine the underlying fouling mechanism: cake filtration, intermediate blocking, pore blocking, or complete blocking [35]. We fitted our data to all four models and determined the goodness of fit using the coefficient of determination (R_fit_^2^) as shown in Equation (13), and the model with the highest R_fit_^2^ was considered the most valid (Table 9). In SCM, we found that a cake layer formed on the 100-kDa and 300-kDa PES membranes and this was the predominant fouling mechanism, whereas the membrane with the highest pore size (100 nm) was subject to both cake filtration and intermediate blocking. In SFM, only the 100-kDa PES membrane showed evidence of significant fouling resistance, which reflected a combination of cake filtration and intermediate blocking effects.

## 4. Discussion

TFF-based concentration/purification is used for the preparation of many virus products, and we therefore investigated its suitability for the production of oncolytic MV given the paucity of recent data on MV recovery and purification [18,21]. Indeed, the only articles dealing with TFF to reduce the volume of MV-containing suspensions were published in the 1970s and 1980s, and further purification was achieved by centrifugation [19,20]. Therefore, to our knowledge, this is the first investigation of TFF as a concentration method for MV, and the first to include the screening of membrane materials, pore sizes/MWCOs, and culture media as process parameters. We focused not only on virus recovery, but also on impurity depletion and the technical characterization of fouling phenomena to achieve a better understanding of the process and its limitations.

A well-designed TFF operation is necessary for fragile biologics such as enveloped viruses and animal cells, and the shear rate and TMP are critical parameters for efficient recovery [29]. MV is very sensitive: it is easily damaged by temperatures exceeding 35 °C, acidic pH and DSP-related shear stress [9,16,17,18]. Therefore, the process conditions for each DSP unit operation must be selected carefully.

We showed that TFF is suitable for the concentration of MV. The recovery of infectious MV was only dependent on the pore size/MWCO, not on the membrane material or the cell culture medium. All membranes with an MWCO ≤ 300 kDa retained infective MV particles, but we were surprised to find that infectious MV was lost when using the 100-nm membrane given the typical size of the MV particle (300–350 nm). Furthermore, we detected no virus in the permeate. Given the large size distribution of MV particles, one explanation is that smaller MV particles enter the membrane pores but become trapped in the pore channels.

Membranes with MWCOs of 100–1000 kDa are generally suitable for virus recovery but they differ in other properties that affect the depletion of impurities. Influenza viruses (100 nm in size) were successfully concentrated using 750-kDa hollow-fiber membranes [24] or 300-kDa PES flat-sheet membranes [23]. Influenza virus-like particles (70–200 nm) were recovered by using membranes with MWCOs of 100–1000 kDa, and in agreement with our results the authors showed that recovery was independent of the membrane material (xRC or PES) [41]. A 300-kDa PES membrane was found to be suitable for the concentration of baculovirus [22], whereas xRC membranes with MWCOs of 300–1000 kDa were suitable for the recovery and concentration of adenoviruses (90–100 nm) [30]. Both 100-kDa and 500-kDa membranes proved efficient for the retention of flaviviruses (~80% recovery), but the higher MWCO was more efficient for the depletion of impurities [42]. However, a more recent study showed that a 300-kDa PES membrane achieved better impurity clearance than the 500-kDa flat-sheet membrane [41].

Most MV studies focus on the recovery of infectious viruses, which are needed for oncolytic therapy. However, the ratio of total to infectious particles (R_T/I_) is rarely reported. This ratio is important because a target threshold is set by the regulatory authorities to avoid the injection of large quantities of inactive particles. Focusing solely on the infectious MV titer, we found that every membrane with a MWCO ≤ 300 kDa achieved almost the full recovery of infective particles. The R_T/I_ of the supernatants we used as feed for the TFF was ~10^4^. In a previous report involving the purification of MV by hydrophobic interaction chromatography, the recovery of infectious MV increased when the R_T/I_ of the feed was higher, and at the highest R_T/I_ of 10^5^ the authors achieved a recovery of ~60%. [21]. Therefore, the outcome of TFF may be influenced by the behavior of the non-infective virus particles. 

We found that the 100-nm membranes adsorbed 40–50% of the total viral RNA, a portion of which represented infective MV particles. For all membranes with a MWCO ≤ 300 kDa, only non-infectious particles and free viral RNA were adsorbed, whereas the infectious particles remained in the retentate. This adsorption behavior was strongly dependent on the medium. In SCM, 10–20% of total viral RNA was lost regardless of the MWCO, whereas the adsorption was much stronger in SFM and was dependent on both the MWCO and the membrane material. Our results indicate that the depletion of total viral RNA/non-infectious virus particles was dependent on the severity of membrane fouling and the resulting saturation of nonspecific binding sites. More nonspecific binding sites were present in the SFM because serum proteins in the SCM mask these sites. This suggests that the presence of some protein in the medium may be beneficial, to prevent nonspecific virus adsorption. Virus adsorption by membranes has not been widely reported. However, TFF with a 100-kDa PES membrane achieved a recovery of 95% for influenza virus, and this was increased to 100% by a post-process membrane wash [43]. Viruses with a similar structure to MV, such as lentiviruses, have been shown to adsorb to membranes in presence of low quantities of protein [44]. Although the lentivirus particles were produced in SCM, the TFF feed was pre-processed by ion exchange chromatography to reduce protein levels. By supplementing the feed with 20 mL of 4 g L^−1^ human serum albumin to block nonspecific binding sites, the authors increased total lentivirus recovery from <10% to 72%, at the cost of introducing a new contaminant into the system [44]. 

In contrast to the results observed with the 100-nm membrane, the adsorption of viruses by membranes with a MWCO ≤ 300 kDa did not cause the loss of infective MV particles. In general, due to its size, MV can be trapped in the pores of the 100-nm membrane or adsorbed on the surface of this and the other membranes, but no particles pass through to the permeate. However, the dominant fouling mechanisms of intermediate blocking and/or cake filtration do not support the theory of pore blocking for the 100-nm membrane. 

Based on our use of the resistance-in-series model, we found that the major source of fouling was not the virus (total or infective) but the protein content of the medium. The protein content of the SFM was 87% lower than that of the SCM due to the absence of serum. The flux when feeding the TFF system with SCM was always lower and the membranes became impermeable over time. The resistances of the 100-nm and 300-kDa membranes, where all impurities normally pass (based on molecular weight), were directly dependent on the total protein content. For example, the total resistance of the 100-nm membrane decreased from 9.1 × 10^11^ (SCM) to 1.3 × 10^11^ (SFM) and overall by ~86%, mirroring the difference in total protein content. The results were similar for the 300-kDa membrane. The difference in resistance when switching from SCM to SFM was much lower for the 100-kDa PES membrane, which had a greater tendency to foul in SFM. This higher degree of fouling suggests that size-exclusion effects were present, and that other molecules were involved in addition to proteins. The expected increase in membrane resistance at lower MWCOs was mirrored by higher flux decay, as previously shown for PES membranes [23,45]. The increase in fouling resistance as the total protein content increased was also supported by the flux decay profile, which was flatter for the SFM. Only one other study has tested the dependency of flux on the culture medium, and this involved the purification of a densonucleosis virus (18–26 nm) [45]. The authors reported that the permeate flux in SFM was lower than that in SCM, which contrasts with our results and indicates the need for careful process evaluation when changing the cell culture medium. 

We observed significant differences in the fouling behavior of the 100-kDa membranes depending on the material. With pure water, the PES membrane was more permeable than the xRC membrane, but in culture medium the xRC membrane was permeable throughout the filtration run and even the higher protein load of the SCM did not lead to any noticeable fouling effects. This low susceptibility to fouling makes this the xRC membrane generally superior for biopharmaceutical processing. For the 100-kDa PES membrane, fouling was not solely dependent on the protein load, because high fouling resistance was observed in both media, suggesting that molecules other than proteins contribute to the fouling of this material.

The efficiency of TFF can be improved by varying the TMP or setting a constant flux. The recovery of rotavirus-like particles was increased by the reduction of TMP to ~86% (down to ~0.35 bar) by using a hollow-fiber module (750 kDa) [31]. This suggests that high TMPs, as used for lentiviruses [44], might reduce the overall recovery. A higher TMP increases the amount of fluid passing along the membrane and therefore the pressure-driven forced movement of fluid on the membrane surface. A similar effect was reported in another study with influenza virus [24]. Here, TFF was carried out in constant flux mode, and a lower constant permeate flux (28 L m^−2^ h^−1^) increased virus recovery to 97%, compared to 44% at the higher flux of 42 L m^−2^ h^−1^. Given that we used a TMP of ~0.2 bar, this effect should be limited in our experimental procedure. However, the variation of TMP and its relationship with MV recovery offers a versatile process option that could be addressed in future experiments. 

Process optimization can follow several directions, so an overall standardized characterization of virus suspensions must be established and clearly defined in terms of product quality for the intended therapeutic application. Given that virus-based therapies are relatively new on the pharmaceutical market, the definition of product quality will be established step by step. However, for complex products such as viral particles, any change in product quality specifications could require significant alterations to the performance and suitability of DSP unit operations.

## 5. Conclusions

Our in-depth analysis of a TFF-based concentration process for oncolytic MV showed that TFF is a robust unit operation for the concentration of infectious MV particles. We considered different membrane materials (PES and xRC), MWCOs (100 nm, 300 kDa and 100 kDa), and two different feeds (SCM and SFM). Regardless of the membrane material and feed, only a MWCO ≤ 300 kDa was suitable for the concentration of infectious MV particles. This is important because the primary aim of any process for the preparation of oncolytic MV is to avoid the loss of infectivity. A second aim is the efficient removal of impurities, which in the case of oncolytic MV also includes non-infectious virus particles. We found that the clearance of non-infectious viruses, total protein, and host cell DNA was strongly dependent on the MWCO and feed composition, revealing a complex dependency between the membrane/feed properties and the outcome of filtration. With regard to fouling, current fouling models do not isolate the effects of single components. It is difficult to gain deeper insight from spiking experiments because this would require pure preparations of only infectious and only non-infectious viruses. We found that purifying a complex product such as MV involves the understanding of coupled interactions between the membrane material, MWCO, and the complex feed (containing cell-culture medium with multiple components, host cell-derived impurities, and infectious and non-infectious MV). Future studies should be conducted using model solutions with MV, and should be extended to the investigation of a diafiltration process and the effect of different impurity levels on the recovery of total and infectious virus particles.

## Figures and Tables

**Table 1:** Different parameters for the four major fouling mechanisms [35].

*m*	Fouling Mechanism	Flux Decline Equation
0	Cake filtration	$J=\frac{J_{0}}{{\left(1+J_{0}^{2}\cdot k_{\mathrm{cf}}\cdot t\right)}^{0.5}}$
1	Intermediate blocking	$J=\frac{J_{0}}{1+J_{0}\cdot k_{\mathrm{ib}}\cdot t}$
1.5	Pore blocking	$J=\frac{J_{0}}{{\left(1+0.5\cdot J_{0}^{0.5}\cdot k_{\mathrm{sb}}\cdot t\right)}^{2}}$
2	Complete blocking	$J=J_{0}\cdot e^{-k_{\mathrm{cb}}\cdot t}$

**Table 2:** Characterization of the MV suspensions based on serum-containing and serum-free media used as the feed in the tangential flow filtration (TFF) experiments. Values are means ± standard derivations (*n* = 8). R_T/I_: ratio of total to infectious MV particles.

Measles Virus In:	Virus Titer (TCID_50_ mL^−1^)	Total Viral RNA (Copies mL^−1^)	R_T/I_ (Copies/TCID_50_)	Proteins (µg mL^−1^)	DNA (ng mL^−1^)
Serum-containing medium (SCM)	3.6 × 10^4^ ± 1.6 × 10^4^	6.5 × 10^8^ ± 7.4 × 10^7^	1.8 × 10^4^	4436.7 ± 623.7	137.9 ± 7.8
Serum-free medium (SFM)	1.0 × 10^5^ ± 4.0 × 10^4^	4.7 × 10^9^ ± 2.4 × 10^9^	4.6 × 10^4^	570.1 ± 78.3	144.9 ± 19.5

**Table 3:** Membrane permeability for total proteins in SCM and SFM. Values are means of two runs (*n* = 2).

MV in	Permeability for Total Proteins			
	PES			xRC
	100 nm	300 kDa	100 kDa	100 kDa
SCM	0.97	0.56	0.35	0.54
SFM	1.00	1.00	1.00	1.00

**Table 4:** Membrane permeability for host cell DNA in SCM and SFM. Values are means of two runs (*n* = 2).

MV in:	Permeability of hcDNA			
	PES			xRC
	100 nm	300 kDa	100 kDa	100 kDa
SCM	0.87	0.51	0.24	0.36
SFM	0.81	0.75	0.36	0.53

**Table 5:** Characterization of the concentrated MV suspensions (final retentate fractions) for SCM or SFM. Values represent the mean of two runs.

Measles Virus in	Virus Titer (TCID_50_ mL^−1^)	Total Viral RNA (Copies mL^−1^)	R_T/I_ (Copies/TCID_50_)	Proteins (mg mL^−1^)	DNA (ng mL^−1^)
**SCM**					
100 nm	1.9 × 10^5^	5.8 × 10^9^	3.1 × 10^4^	7.3	243.0
300 kDa	2.4 × 10^6^	8.2 × 10^9^	3.3 × 10^3^	23.1	896.7
100 kDa (PES)	2.9 × 10^6^	8.7 × 10^9^	3.0 × 10^3^	46.1	1045.7
100 kDa (xRC)	2.1 × 10^6^	8.5 × 10^9^	4.1 × 10^3^	42.4	1127.1
**SFM**					
100 nm	8.2 × 10^5^	2.4 × 10^10^	2.9 × 10^4^	1.1	346.6
300 kDa	2.9 × 10^6^	4.2 × 10^10^	1.4 × 10^4^	1.7	494.9
100 kDa (PES)	4.2 × 10^6^	2.9 × 10^10^	6.8 × 10^3^	1.8	1547.9
100 kDa (xRC)	3.9 × 10^6^	1.6 × 10^10^	4.0 × 10^3^	1.3	1229.8

**Table 6:** Productivity of tangential flow filtration for the purification of MV in SCM and SFM at a transmembrane pressure of ~0.2 bar. Values are means of two runs (*n* = 2).

MV in:	Productivity (L m^−2^ h^−1^)			
	PES			xRC
	100 nm	300 kDa	100 kDa	100 kDa
SCM	66.6	44.4	27.6	50.1
SFM	134.7	95.7	40.8	59.9

**Table 7:** Viscosities of the permeate fractions in SCM and SFM and pure water at 20 °C. Values are means ± standard deviations (*n* = 3).

Measles Virus in	100 nm/300 kDa (mPa s)	100 kDa (PES/xRC) (mPa s)	Pure Water (mPa s)
SCM	1.29 ± 0.05	1.11 ± 0.06	0.96 ± 0.02
SFM	1.03 ± 0.01		

**Table 8:** Flux recovery for each membrane and cell culture medium combination. After filtration, the membranes were flushed with MilliQ water and the flux was measured. The flux recovery is expressed as water flux after MV filtration in relation to the pure water flux with the clean membrane, indicating the degree of fouling. Values are means of two runs (*n* = 2).

MV in	Relative Water Flux after Filtration			
	PES			xRC
	100 nm	300 kDa	100 kDa	100 kDa
SCM	0.60	0.53	0.45	0.90
SFM	0.89	0.86	0.59	0.94

**Table 9:** R_fit_^2^ values for the fitting of experimental data to the four fouling models [35] in Matlab (*n.d.* – not determined).

Material	Cut-Off	Cake Filtration		Intermediate Blocking		Pore Blocking		Complete Blocking	
		SCM	SFM	SCM	SFM	SCM	SFM	SCM	SFM
PES	100 nm	0.98	*n.d.*	0.98	*n.d.*	0.97	*n.d.*	0.95	*n.d.*
	300 kDa	0.96	*n.d.*	0.95	*n.d.*	0.95	*n.d.*	0.94	*n.d.*
	100 kDa	0.97	0.98	0.96	0.98	0.96	0.97	0.95	0.96

## Abbreviations

PES	Polyether sulfone
xRC	Cross-linked regenerated cellulose
A	Membrane area (m^2^)
V	Permeate volume (m^3^)
t	Time (s)
J	Flux (m^3^ m^−2^ s^−1^)
µ	Dynamic viscosity (N m^−2^ s)
$p_{f}$	Feed pressure (Pa)
$p_{r}$	Retentate pressure (Pa)
$p_{p}$	Permeate pressure (Pa)
TMP	Transmembrane pressure (Pa)
$R_{\mathrm{total}}$	Total membrane resistance (m^−1^)
$R_{m}$	Membrane resistance (m^−1^)
$R_{f}$	Fouling resistance (m^−1^)
SCM	Serum-containing medium
SFM	Serum free medium,
HCP	Host cell proteins
hcDNA	Host cell DNA
L_P_	Hydraulic permeability (L m^−2^ h^−1^ bar^−1^)
J_w_, J_0_	Pure water flux (L m^−2^ h^−1^)
α	Permeability
m	Fouling constant (-)
k	Resistance coefficient
c	Concentration (feed, permeate, retentate)
c_0_	Initial concentration
c_i_	Concentration at a specific concentration factor (cf)
cf	Concentration factor (-)
$\hat{y_{i}}$	y-value of the model function
$y_{i}$	y-value of the experimental data
k_cf_	Fouling constant – cake filtration
k_ib_	Fouling constant – intermediate blocking
k_sb_	Fouling constant – standard blocking
k_cb_	Fouling constant – complete blocking
Re	Reynolds number (-)
SSE	Sum square error
R_fit_^2^	Coefficient of determination
MWCO	Molecular weight cut-off (kDa)
*n.d.*	not determined

## Appendix A

The sequence of the MV N-protein used for quantification by RT-qPCR is 5′-ACUUGCUCUGCUGGGCCCGGUUUCUCUGUAGCUCUCCCUGGCUUCUCCUCGACUCUGUUUGACCCUCCUAUCUUCCUUGCCCCCCAAUCUCGGUAGCUCAUUCUCACUUUGAUCACCGUGUAGAAAUGAUACUUGGGCUUGUCUGGGUCCAACCGCUCUACUGAUCUUGUCCUCAGUAGUAUGCAUUGCAAUCUCUGAAACAAGCCAUGCAUCCUCGGCAGUGAUACCGAGUUCAGAUCCAAUGUGGAACUGACCUUUCCAGCUGACCUCCUUACCAUCUCUUGCCCUAAUCUAAAAUA-3′.

## References

[1] Chiocca E.A., Rabkin S.D. (2014). Oncolytic viruses and their application to cancer immunotherapy. Cancer Immunol. Res..

[2] Fielding A.K. (2005). Measles as a potential oncolytic virus. Rev. Med. Virol..

[3] Russell S.J., Whye Peng K. (2009). Measles virus for cancer therapy. Curr. Top. Microbiol. Immunol..

[4] Galanis E., Atherton P.J., Maurer M.J., Knutson K.L., Dowdy S.C., Cliby W.A., Haluska P., Long H.J., Oberg A., Aderca I. (2015). Oncolytic measles virus expressing the sodium iodide symporter to treat drug-resistant ovarian cancer. Cancer Res..

[5] Weiss K., Gerstenberger J., Salzig D., Mühlebach M.D., Cichutek K., Pörtner R., Czermak P. (2015). Oncolytic measles viruses produced at different scales under serum-free conditions. Eng. Life Sci..

[6] Grein T.A., Schwebel F., Kress M., Loewe D., Dieken H., Salzig D., Weidner T., Czermak P. (2017). Screening different host cell lines for the dynamic production of measles virus. Biotechnol. Prog..

[7] Grein T.A., Loewe D., Dieken H., Weidner T., Salzig D., Czermak P. (2019). Aeration and Shear Stress Are Critical Process Parameters for the Production of Oncolytic Measles Virus. Front. Bioeng. Biotechnol..

[8] Grein T.A., Loewe D., Dieken H., Salzig D., Weidner T., Czermak P. (2018). High titer oncolytic measles virus production process by integration of dielectric spectroscopy as online monitoring system. Biotechnol. Bioeng..

[9] Weiss K., Salzig D., Röder Y., Gerstenberger J., Mühlebach M.D., Cichutek K., Pörtner R., Czermak P. (2013). Influence of process conditions on measles virus stability. Am. J. Biochem. Biotechnol..

[10] Weiss K., Salzig D., Mühlebach M.D., Cichutek K., Pörtner R., Czermak P. (2012). Key parameters of Measles virus production for oncolytic virotherapy. Am. J. Biochem. Biotechnol..

[11] Wright J.F. (2014). Product-Related Impurities in Clinical-Grade Recombinant AAV Vectors: Characterization and Risk Assessment. Biomedicines.

[12] Kramberger P., Urbas L., Štrancar A. (2015). Downstream processing and chromatography based analytical methods for production of vaccines, gene therapy vectors, and bacteriophages. Hum. Vaccines Immunother..

[13] Champion K., Madden H., Dougherty J., Shacter E. (2005). Defining Your Product Profile and Maintaining Control Over It, Part 2: Challenges of Monitoring Host Cell Protein Impurities: Challenges of Monitoring Host Cell Protein Impurities. BioProcess Int..

[14] WHO Expert Committee on Biological Standardization, World Health Organization, Ebrary, Inc. (2007). WHO Expert Committee on Biological Standardization.

[15] Loughney J.W., Lancaster C., Ha S., Rustandi R.R. (2014). Residual bovine serum albumin (BSA) quantitation in vaccines using automated Capillary Western technology. Anal. Biochem..

[16] Daikoku E., Chizuko M., Kohno T., Sano K. (2007). Analysis of Morphology and Infectivity of Measles Virus Particles. Bull. Osaka Med Coll..

[17] Loewe D., Häussler J., Grein T.A., Dieken H., Weidner T., Salzig D., Czermak P. (2019). Forced degradation studies to identify critical parameters for Measles virus purification. Viruses.

[18] Sviben D., Forčić D., Kurtović T., Halassy B., Brgles M. (2016). Stability, biophysical properties and effect of ultracentrifugation and diafiltration on measles virus and mumps virus. Arch. Virol..

[19] Udem A.S. (1984). Measles virus: Conditions for the propagation and purification of infectious virus in high yield. J. Virol. Methods.

[20] Bellini W.J., Trudgett A., McFarlin D.E. (1979). Purification of measles virus with preservation of infectivity and antigenicity. J. Gen. Virol..

[21] Sviben D., Forcic D., Ivancic-Jelecki J., Halassy B., Brgles M. (2017). Recovery of infective virus particles in ion-exchange and hydrophobic interaction monolith chromatography is influenced by particle charge and total-to-infective particle ratio. J. Chromatogr. B Analyt. Technol. Biomed. Life Sci..

[22] Michalsky R., Passarelli A.L., Pfromm P.H., Czermak P. (2009). Purification of the baculovirus Autographa californica M nucleopolyhedrovirus by tangential flow ultrafiltration. Desalination.

[23] Wickramasinghe S.R., Kalbfuss B., Zimmermann A., Thom V., Reichl U. (2005). Tangential flow microfiltration and ultrafiltration for human influenza A virus concentration and purification. Biotechnol. Bioeng..

[24] Kalbfuss B., Genzel Y., Wolff M., Zimmermann A., Morenweiser R., Reichl U. (2007). Harvesting and concentration of human influenza A virus produced in serum-free mammalian cell culture for the production of vaccines. Biotechnol. Bioeng..

[25] Nehring D., Gonzalez R., Pörtner R., Czermak P. (2004). Experimental and modeling study of a membrane filtration process using ceramic membranes to increase retroviral pseudotype vector titer. J. Membr. Sci..

[26] Negrete A., Pai A., Shiloach J. (2014). Use of hollow fiber tangential flow filtration for the recovery and concentration of HIV virus-like particles produced in insect cells. J. Virol. Methods.

[27] Vicente T., Peixoto C., Carrondo M.J.T., Alves P.M. (2009). Purification of recombinant baculoviruses for gene therapy using membrane processes. Gene Ther..

[28] Grein T.A., Michalsky R., Czermak P. (2014). Virus separation using membranes. Methods Mol. Biol..

[29] Maiorella B., Dorin G., Carion A., Harano D. (1991). Crossflow microfiltration of animal cells. Biotechnol. Bioeng..

[30] Nestola P., Martins D.L., Peixoto C., Roederstein S., Schleuss T., Alves P.M., Mota J.P.B., Carrondo M.J.T. (2014). Evaluation of novel large cut-off ultrafiltration membranes for adenovirus serotype 5 (Ad5) concentration. PLoS ONE.

[31] Peixoto C., Sousa M.F.Q., Silva A.C., Carrondo M.J.T., Alves P.M. (2007). Downstream processing of triple layered rotavirus like particles. J. Biotechnol..

[32] Opdensteinen P., Clodt J.I., Müschen C.R., Filiz V., Buyel J.F. (2018). A Combined Ultrafiltration/Diafiltration Step Facilitates the Purification of Cyanovirin-N from Transgenic Tobacco Extracts. Front. Bioeng. Biotechnol..

[33] Czermak P., Grzenia D.L., Wolf A., Carlson J.O., Specht R., Han B., Wickramasinghe S.R. (2008). Purification of the densonucleosis virus by tangential flow ultrafiltration and by ion exchange membranes. Desalination.

[34] Hensgen M.I., Czermak P., Carlson J.O., Wickramasinghe S.R. (2010). Purification of Minute Virus of Mice using high performance tangential flow filtration. Desalination.

[35] Hermia J. (1982). Constant pressure blocking filtration laws—Application to power-law non-Newtonian fluids. Chem. Eng. Res. Des..

[36] Kärber G. (1931). Beitrag zur kollektiven Behandlung pharmakologischer Reihenversuche. Arch. Exp. Pathol. Pharmakol..

[37] Reed L.J., Muench H. (1938). A simple method of estimating fifty per cent endpoints. Am. J. Epidemiol..

[38] Baekelandt V., Eggermont K., Michiels M., Nuttin B., Debyser Z. (2003). Optimized lentiviral vector production and purification procedure prevents immune response after transduction of mouse brain. Gene Ther..

[39] Rodrigues T., Carrondo M.J.T., Alves P.M., Cruz P.E. (2007). Purification of retroviral vectors for clinical application: Biological implications and technological challenges. J. Biotechnol..

[40] Tuschong L., Soenen S.L., Blaese R.M., Candotti F., Muul L.M. (2002). Immune response to fetal calf serum by two adenosine deaminase-deficient patients after T cell gene therapy. Hum. Gene Ther..

[41] Carvalho S.B., Silva R.J.S., Moleirinho M.G., Cunha B., Moreira A.S., Xenopoulos A., Alves P.M., Carrondo M.J.T., Peixoto C. (2019). Membrane-Based Approach for the Downstream Processing of Influenza Virus-Like Particles. Biotechnol. J..

[42] Mundle S.T., Giel-Moloney M., Kleanthous H., Pugachev K.V., Anderson S.F. (2015). Preparation of pure, high titer, pseudoinfectious Flavivirus particles by hollow fiber tangential flow filtration and anion exchange chromatography. Vaccine.

[43] Nayak D.P., Lehmann S., Reichl U. (2005). Downstream processing of MDCK cell-derived equine influenza virus. J. Chromatogr. B Analyt. Technol. Biomed. Life Sci..

[44] Bandeira V., Peixoto C., Rodrigues A.F., Cruz P.E., Alves P.M., Coroadinha A.S., Carrondo M.J.T. (2012). Downstream processing of lentiviral vectors: Releasing bottlenecks. Hum. Gene Ther. Methods.

[45] Grzenia D.L., Carlson J.O., Czermak P., Han B., Specht R.K., Wickramasinghe S.R. (2006). Purification of densonucleosis virus by tangential flow ultrafiltration. Biotechnol. Prog..

